# Neural circuit plasticity transforms infant neglect into maternal care

**DOI:** 10.21203/rs.3.rs-5983736/v1

**Published:** 2025-02-26

**Authors:** Chloe J. Bair-Marshall, Naomi L. Cassel, Ayat A. Agha, Malak Bkhiet, Robert C. Froemke

**Affiliations:** 1 Neuroscience Institute, New York University School of Medicine, New York, NY 10016 USA.; 2 Department of Otolaryngology, New York University School of Medicine, New York, NY 10016, USA.; 3 Department of Neuroscience and Physiology, New York University School of Medicine, New York, NY 10016, USA.; 4 Center for Neural Science, New York University, New York, NY 10003, USA.; 5 Barnard College, New York, NY 10027, USA.

## Abstract

Infants in distress evoke strong emotional responses in adults, which help to elicit caretaking behaviors from parents to ensure infant needs are met^1–3^. However, neonatal care can also be challenging, and interactions with infants can lead to stress and negative affect even in potential caregivers^4–7^. Child neglect and maltreatment rates in human populations make it important to understand the neural mechanisms of regulating negative emotions and stress in the parental brain^8–10^. Here we show how rapid plasticity in female mouse central amygdala (CeA) transforms infant aversion into attentiveness after initial pup experience. Projections from CeA to locus coeruleus (CeA→LC) were strongly activated upon initial pup contact leading to pup aversion. CeA→LC pup responses were reduced with parental experience and down-regulating CeA→LC activity led to less aversion. Oxytocin signaling in central amygdala was required to switch pup aversion to attention, inducing rapid long-term depression of excitatory inputs onto projection neurons. CeA→LC projectors released the stress hormone corticotropin-releasing factor onto LC neurons, modulating phasic firing to regulate attention. This circuit organization enables local CeA computations of pup valence to be broadcast throughout the brain by the LC central arousal system, leading to appropriate pup-directed behaviors depending on adult state.

## Main

Most mammals are born without the ability to feed, protect, or transport themselves, and must rely entirely on parental adults to survive during early life. As a result, biological parents and other unrelated caretaking adults (‘alloparents’) experience strong emotional responses to cues that indicate the physiological needs or distress of infants^6,11^. In experienced parents, these responses trigger approach towards distressed infants and lead to caretaking^2,12–16^. However, in infant-naïve animals, encounters with young animals or strong responses evoked by neonatal distress can instead lead to negative affect^6,7,17,18^, neglect or avoidance, and in some cases infant mortality^10,18–20^. Negative responses to infants also have significant consequences for adult mental health and well-being, underscored by the prevalence and severity of peri-partum disorders in humans^10,19,21^. Thus, it is critical to understand the neural circuits that determine emotional responses to social stimuli such as infants, as well as the mechanisms that enable these circuits to be rapidly modified in new parents.

Regions of the amygdala, and the central nucleus CeA in particular, are thought to process the emotional valence of different stimuli and experiences^22–24^. However, most studies of amygdala threat processing involve responses to conditioned stimuli instead of potential natural stressors such as other adults or infants. One peculiar biological feature of CeA is that this region expresses oxytocin receptors at much higher levels than most other brain areas^25,26^, but it is unclear why CeA would be specialized for maternal hormone signaling. Recently our lab and others have described how central oxytocin signaling helps nulliparous (‘virgin’) female mice learn to become alloparents via rapid plasticity in the auditory cortex for the sounds of infant distress calls^27,28^. We wondered if oxytocin signaling might therefore also help enable changes in other parts of the mammalian brain such as the CeA, given the complexities of parenting and the numerous adaptations and behavioral changes required to quickly produce adequate caregiving behavior.

### Brief experience with pups transforms aversion into attentiveness

**Figure 1.**
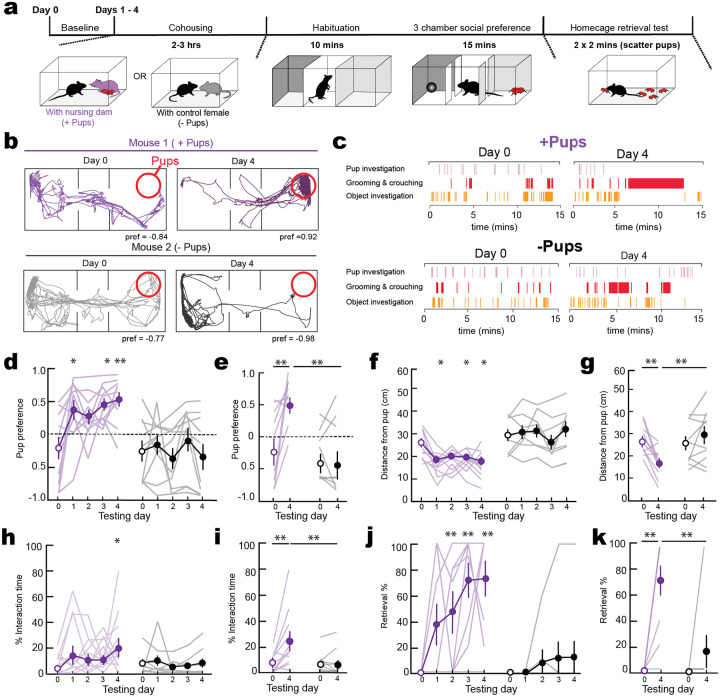
Cohousing with nursing dam alters pup-approach dynamics **a-c,** Cohousing and behavioural protocols. Schematic (**a**), example video tracking (**b**) and time course (**c**) during 3-chamber pup approach assay from representative animals cohoused with nursing dam and litters (+pups, purple) or with control adult females (−pups, grey). **d,e,** Virgin females cohoused with nursing dams and litters (purple), but not control adults (black), significantly increased pup-related approach behaviors. Pup preference scores across all cohousing days (**d**; cohoused +pups, day 0 compared with day 1–4: p=0.039, p=0.115, p=0.018, p=0.006) and on day 0 vs 4 (**e**; +pups, day 0 vs 4: p=0.001; day 4, +pups vs −pups: p=0.0007). 2-way ANOVAs, corrected for multiple comparisons. Lines, individual mice; circles, mean±s.e.m. (*, p<0.05; **, p<0.01). **f,g,** Average distance from pups across all cohousing days (**f**; cohoused +pups, day 0 compared with day 1–4: p=0.013, p=0.180, p=0.008, p=0.007) and on day 0 vs 4 (**g**; +pups, day 0 vs 4: p= 0.0007; day 4, +pups vs −pups: p=0.0001). **h,i,** Proportion of total time interacting with pups across all cohousing days (**h**; cohoused + pups, day 0 compared with day 1–4: p=0.241, p=0.196, p=0.421, p=0.035) and on day 0 vs 4 (**I**; + pups, day 0 vs 4: p=0.0006, day 4; +pups vs −pups p=0.006). **j,k,** Percentage of total pups retrieved across all days (**j**; cohoused + pups, day 0 compared with day 1–4: p=0.123, p=0.010, p=0.0005, p=0.0002) and on day 0 vs 4 (**k**; + pups day 0 vs 4: p<0.0001, day 4; + pups vs - pups p<0.0001). N=11 cohoused (+pups), N=10 control (−pups).

We first aimed to establish a robust behavioral framework for quantifying the transition from pup aversion to attentiveness in first-time parental mice. Previous studies have shown that cohousing a virgin female with a dam and pups leads to alloparenting by the virgin over hours to days^27,29^. We studied this process in adult female virgins raised without exposure to dams or pups after weaning (Fig. 1a). One cohort of virgin females was cohoused with a nursing dam and litter (denoted as +pups) for 2–3 hours daily over four days, and another cohort cohoused with a control adult female (denoted as −pups). After each cohousing session, we examined each animal in a 3-chamber social preference assay. We analyzed time in the side containing pups vs the side containing a novel object to compute a pup preference score between −1.0 if 100% of time was spent in the novel object chamber and 1.0 if 100% of time was spent in the chamber with pups (Fig. 1a–d). We first tested animals at baseline (‘Day 0’) before cohousing and found that naïve virgin females initially avoided pups (Fig. 1d,e; purple, pup preference score on day 0 for mice cohoused with dam and pups (+pups): −0.24±0.17; black, pup preference score on day 0 for mice cohoused with adult female but no pups (−pups): −0.27±0.16). However, just a single 2–3 hour cohousing session led to a substantial switch in virgin side preference to the chamber containing pups, maintained throughout all four days of testing (Fig. 1d, purple +pups, day 1: 0.35±0.15, p=0.039, day 2: 0.24±0.11, p= 0.115, day 3: 0.44±0.09, p=0.018, day 4: 0.51±0.11, p=0.006, 2-way ANOVA with Dunnett’s multiple comparison correction, N=11 virgins cohoused with dam and pups).

Conversely, animals cohoused with another adult female did not change pup aversion over days (Fig. 1d, black, –pups, day 1 pup preference score: −0.17±0.14, day 2: −0.39±0.16, day 3: −0.09±0.18, day 4: −0.36±0.20, p>0.05, 2-way ANOVA with Dunnett’s multiple comparison correction, N=10 virgins housed with control female). Reduced pup aversion and increased attention was measured as a decreased distance from pups (Fig. 1f,g; +pups, day 0: 25.36±1.94 cm, day 4: 15.57±1.96 cm, N=11, p=0.007;–pups, day 0: 28.0±2.94 cm, day 4: 31.81±3.77 cm, N=10, p=0.15, 2-way ANOVA with Fisher’s test), and increased proportional amount of time interacting with pups (Fig. 1h,i; +pups: day 0: 7.77±2.31% of total test time, day 4: 24.48±6.96%, p=0.0006, N=11;–pups, day 0: 6.77±2.55%, day 4: 6.50±3.30%, p=0.98, N=10, 2-way ANOVA with Fisher’s test). We also observed that these brief sessions of daily pup exposure were sufficient for onset of alloparenting behavior in the virgin females, who began retrieving pups when tested with a pup separated from the nest. Pup retrieval did not emerge in females cohoused with just another adult (Fig. 1j,k, +pups, retrieval on day 0: 0.0±0.0%, pup retrieval on day 4: 74.5±11.5%, p<0.0001, N=11; −pups, pup retrieval on day 0: 0.0±0.0%, pup retrieval on day 4: 21.0±12.5%, p>0.05, N=10, 2-way ANOVA with Fisher’s test).

**Extended Data Figure 1.**
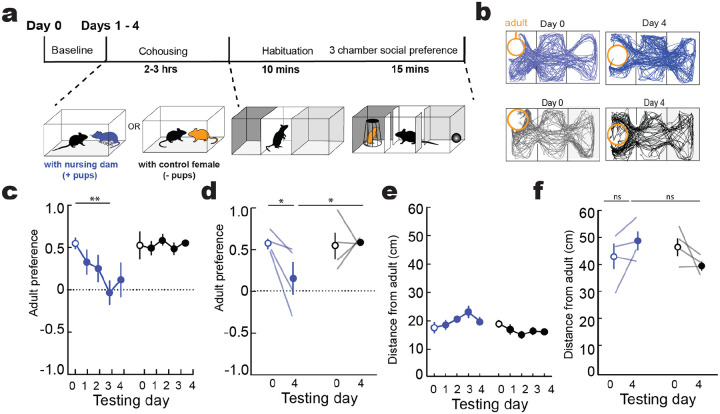
Cohousing did not increase preference for unfamiliar adults **a,** Experimental timeline. **b,** Video tracking examples of virgins exploring an arena with a restrained novel adult after 0 or 4 sessions of cohousing with a dam and litter (blue) or control adult female (grey). **c,d**, Cohousing with nursing dam does not increase preference for adults. Adult preference score for cohoused virgins over 4 days (**c**) or comparing day 0 to day 4 (**d**) (cohoused: day 0 versus day 4; p=0.044, day 4 cohoused vs non-cohoused: p=0.034, N=4 both groups). Lines, individual animals; circles, mean ± s.e.m. **e,** average distance from adult over 4 days of cohousing (e) or comparing between day 0 and 4 (**f**) (cohoused: day 0 versus day 4; p=0.186, day 4 cohoused vs non-cohoused: p=0.072, N=4 both groups, Fisher’s test).

This increase in pup preference did not result from a general increase of sociability. In a separate cohort of virgin females cohoused daily with a dam and pups, we followed cohousing with a social preference test for an unfamiliar adult female instead of pups. Cohousing did not increase social side preference over days (Extended Data Fig. 1). These data show that virgin females are initially pup-averse, and that short bouts of cohousing elicit a specific reversal in the preference for pups resulting in a behavioral switch from avoidance to approach.

### CeA→LC projection-specific plasticity of pup responses after parental experience

The switch in pup preference suggests that cohousing altered the affective valence of pups for virgin female mice. In humans, amygdala activity is associated with affective responses to infant distress and quality of parental attentiveness^6^. Among amygdala subregions, CeA is believed to compute behavioral responses to salient stimuli across the valence continuum^23^. CeA also projects extensively throughout the brain including to areas associated with maternal and social behaviors^24,30^. Additionally, CeA exhibits a high level of oxytocin receptor expression^25,26^, and oxytocin release in CeA has been shown to reduce fear and anxiety related behaviors^31,32^. These features suggest that CeA is a critical node in the neural circuit for social-maternal behavior, but whether and how CeA circuits encode social stimuli has so far received little attention.

**Figure 2.**
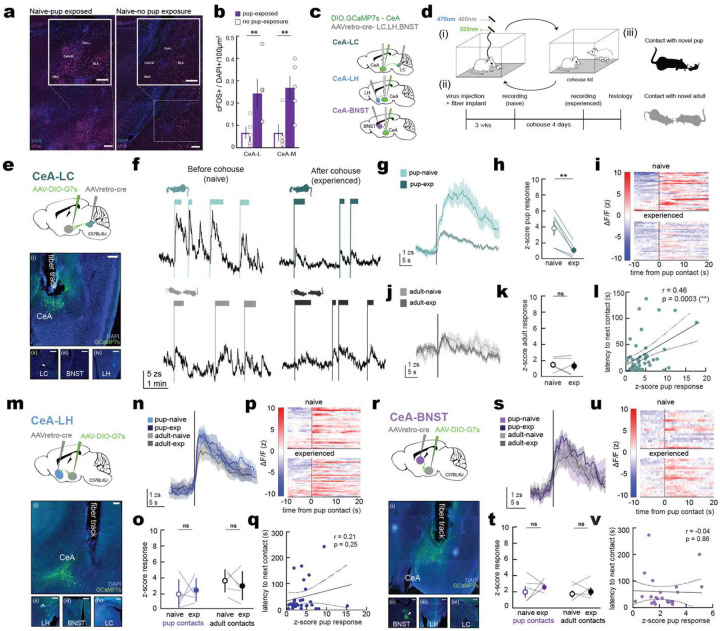
Cohousing causes reduction of CeA→LC pup-related activity **a,b,** Pup-naïve females show activated central amygdala subdivisions after pup exposure. Representative histological images showing c-Fos (magenta) and DAPI (blue) expression in the amygdala (inset) of pup-naïve virgin female mice after 15 minutes of pup exposure (left) compared to control (right). **(a)**. Pup-exposed animals have higher ratios of c-Fos+/DAPI+ cells per 100 μm^2^ of tissue in both medial and lateral subdivisions of CeA **(b)**. Scale, 200 μm. **c,** Schematic of projection-specific targeting of projections to LC, LH, and BNST. **d,** Experimental schematic (i) and timeline (ii) describing recording protocols before and after 4-days of cohousing. Virgins were presented with either unfamiliar pups or adults (iii). **e-l,** CeA projections to LC undergo experience-dependent reduction in pup, but not adult-related activity after cohousing. **e,** Schematic (top) and representative histology bottom of photometry recording of CeA projections to LC where axons are visible in LC (ii, closed arrow) but not in other projection areas (iii, iv). **f,** Example traces from photometry recording of CeA-LC neurons during pup interactions (top) and adult interactions (bottom). **g,** Peri-event time histograms (PETHs) of activity during pup-interactions of the same animals before (light) and after (dark) cohousing. Shaded line, mean ± s.e.m. **h,** Average change in z-scored GCaMP7s signal in response to pups before (naïve, light green) and after (experienced, dark green) cohousing (N=6, p=0.0003, two-way ANOVA). Lines, individual animals; circles, mean ± s.e.m. **i,** Heatmap of z-scored GCaMP7s signal during individual pup-interaction trials. **j,** PSTH of adult responses. **k,** Average z-score responses to adults (N=5, p=0.754, two-way ANOVA). **l,** Pearson correlation of z-scored pup-response in CeA-LC cells with latency to approach pup on subsequent trial (n=53 trials from N=6 animals; r: 0.46, p=0.0003). **m-v,** CeA projections to LH and BNST do not change with cohousing. **m,** schematic (top) and histology (bottom) showing photometry recording of CeA projections to LH (ii, closed arrow) with some axons in BNST (iii, open arrow) but no overlap with LC projections (iv). PSTHs (**n**) and z-scored average responses (**o**) before and after cohousing in CeA-LH population. **p,** heatmap of individual pup exposure trials for CeA-LH projections. **q,** Pearson correlation of z-scored pup-response with latency to approach pup on subsequent trial (n=32 trials from N=5 animals; r: 0.208, p=0.253). **r,** schematic (top) and histology (bottom) showing photometry recording of CeA projections to BNST (ii, closed arrow) with some axons in LH (iii, open arrow) but no overlap with LC projections (iv). PSTHs (**s**) and z-scored average responses (**t**) before and after cohousing in CeA-LH population. **u,** heatmap of individual pup exposure trials for CeA-BNST projection. **v,** Pearson correlation of z-scored pup-response with latency to approach pup on subsequent trial (n=26 trials from N=5 animals; r: −0.037, p=0.858).

We first examined expression of the immediate-early gene cFos in virgin female mice exposed to pups. More cFos+ cells were observed in both the lateral and medial CeA of pup-exposed virgins (Fig. 2a,b; CeA-M, pup exposed: 0.066±0.026 c-Fos+/DAPI+ cells per 100 μm^2^ of tissue; no pup exposure: 0.27±0.056 cells, p=0.0021; CeA-L, pup exposed: 0.07±0.036 cells, no pup exposure: 0.30±0.035 cells, p=0.0008, N=5 per group). We then used photometry to image activity of CeA subpopulations with different downstream projection targets. Previous studies using genetic markers to access subpopulations of CeA neurons showed that genetically-defined subtypes of CeA neurons generally overlap in terms of projection area or location within CeA^30^. We therefore targeted subpopulations based on projections by injecting retrograde Cre virus in projection targets and another injection of Cre-dependent GCaMP7s in CeA (Fig. 2c). We focused on three main targets: lateral hypothalamus (LH), the bed nucleus of the stria terminalis (BNST), and brainstem LC, due to the importance of these brain areas for social and parental behavior^12,18,33–35^.

**Extended Data Figure 2.**
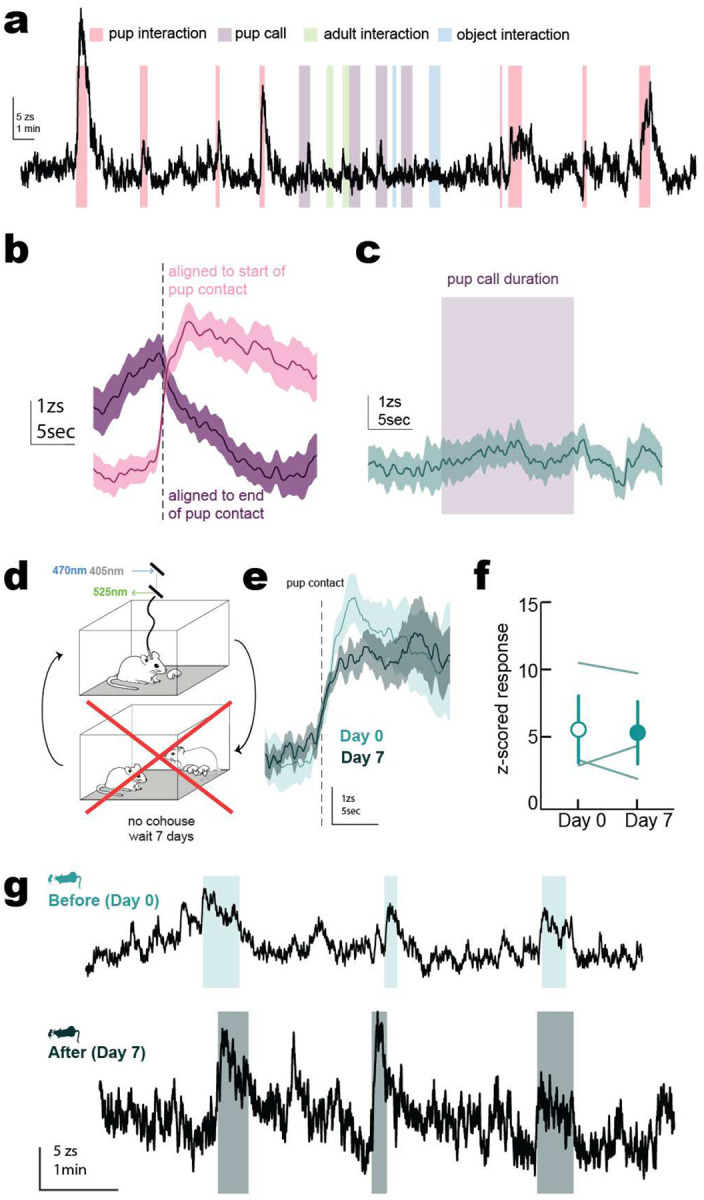
Control experiments for CeA→LC fiber photometry **a**, Example full length fiber photometry recording of CeA→LC neurons during pup interactions (red), pup call presentations via speaker (purple), adult interactions (green), and interactions with a novel object (blue). **b**, CeA→LC activity aligned to either the start of pup interactions (pink) and the end of pup interactions (purple) show that activity started at contact and lasted for the duration of pup investigation. **c**, CeA→LC neuron activity did not increase during presentation of pup calls. **d-g**, CeA→LC pup responses were unchanged if cohousing did not occur. **d**, Schematic of protocol where virgins wait 7 days between recordings without cohousing. **e**, Mean responses with shaded ± s.e.m of activity aligned to onset of pup interactions on day 0 (light green) and day 7 responses (dark green). **f**, Mean change in z-score in response to pups on day 0 and day 7 (p=0.8083, Student’s two-tailed paired t-test). Lines, individual animals; circles, mean ± s.e.m. **g**, Example CeA-LC recording in same animal at day 0 (top) and day 7 (bottom) if cohousing does not occur.

We imaged activity of these three main CeA projections during interactions with unfamiliar neonates and unfamiliar adults in the same animals both before (naïve) and after (experienced) cohousing (Fig. 2d). Interactions with pups or adults led to transient increases in activity for each of these three CeA projection types, suggesting that CeA neurons readily respond to a range of social stimuli. However, only the LC projecting population (CeA→LC) showed specific reductions in pup-evoked activity after cohousing and switch from pup aversion to attention (Fig. 2e–g). CeA→LC neurons initially had large peaks when virgins first approached and interacted with pups, but amplitudes of CeA→LC responses were much smaller in these same animals after cohousing experience with pups and onset of alloparenting (Fig. 2g,h; ‘naïve’, light green, pup response: 3.84±0.85 z-score change; ‘experienced’, dark green, pup response: 0.96±0.33 z-score change, N=6 mice, p=0.0003). Pup-evoked transients could last for tens of seconds (Fig. 2i), generally persisting until the end of the interaction (Extended Data Fig. 2a,b). Similar transients were not observed in response to adults (Fig. 2j,k; ‘naïve’, light grey, adult response: 1.47±0.37 z-score change, ‘experienced’, dark grey, adult response: 1.29±0.50 z-score change, N=5 mice, p=0.754), and not dampened after cohousing just with another adult (Extended Data Fig. 2). Responses were not evoked by distress calls alone, as playback of ultrasonic pup distress calls did not evoke large transients (Extended Data Fig. 2a,c). We noticed that after initial pup investigation, naïve virgins usually avoided pups for seconds to minutes afterward. Examining the amplitude of CeA→LC responses showed that the amplitude of this signal was correlated with latency to the next investigation, i.e., the duration of pup aversion, with larger CeA→LC transients leading to longer pauses between pup interactions (Fig. 2l, n=53 events, r: 0.46, p=0.0003).

In contrast to the specificity and experience-dependent changes of CeA→LC transients, responses of other CeA outputs were not modulated by experience and unrelated to pup aversion. CeA→LH projections (Fig. 2m–q) and CeA→BNST projections (Fig. 2r–x) were equivalent in strength for interactions with adults or pups (Fig. 2p, CeA→LH projections, responses to pups when naïve: 1.90±0.82 z-score change, pup responses when experienced: 2.46±0.64 z-score change, p=0.568; response to adult animals when naïve: 3.59±0.61 z-score change, responses to adults when experienced: 2.96±0.78 z-score change, p=0.115, N=5; Fig. 2v, CeA→BNST projections, responses to pups when naïve: 2.71±0.73 z-score change, responses to pups when experienced:= 2.94±0.33 z-score change, p=0.802, responses to adults when naïve: 2.56±0.63 z-score change, responses to adults when experienced: 2.69 ±0.45 z-score change, p=0.885, N=5).

Magnitude of single transients was also uncorrelated with the duration of pup aversion CeA→LH projections (Fig. 2r, n=32 events, r: 0.208, p=0.253) and CeA→BNST projections (Fig. 2x, n=26 events, r: −0.037, p=0.858). Our results show that while many output cells and pathways from the CeA respond to social stimuli, only outputs to LC are modified after pup experience: initially high in magnitude when animals are pup-averse and dampened in amplitude when animals are parental.

### CeA→LC activity drives pup avoidance

**Figure 3.**
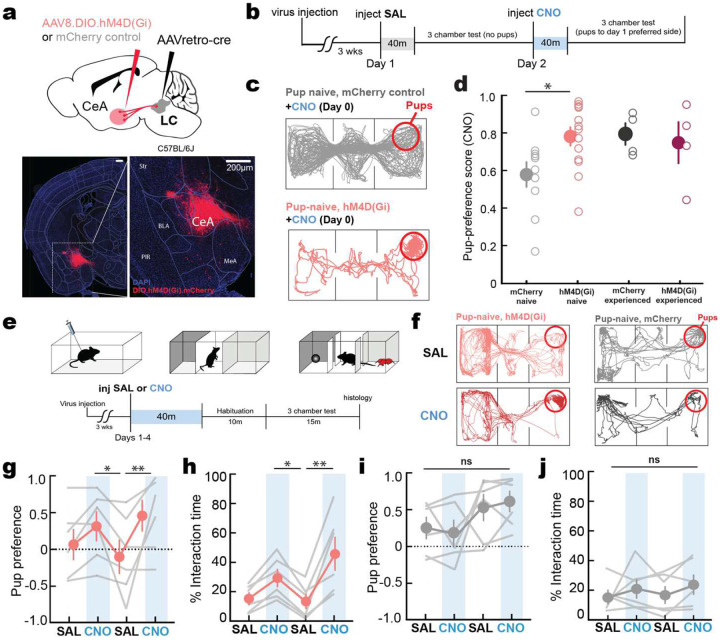
Chemogenetic inhibition of CeA to LC neurons reduces pup avoidance **a,** Schematic (top) and representative histology (bottom). Scale, 200 μm. **b,** Timeline of behavioral testing. **c,d,** Inhibiting CeA-LC neurons reduced baseline pup aversion. Video tracking examples **(c)** and quantification of pup-preference score **(d)** reveals increased baseline preference for pups in pup-naïve virgins expressing inhibitory DREADDs in CeA-LC neurons (N=10 mCherry-naïve mice, N=13 Gi-naïve, N=4 mCherry-experienced, N= 4 Gi-experienced; mCherry-naïve versus Gi-naïve: p=0.016, Fisher’s test). Lines, open circles, individual mice; filled circles, mean ± s.e.m. **e-j,** Inhibiting CeA-LC neurons reduces pup avoidance over days. **e,** schematic and illustration of multi-day chemogenetic experiment. Video tracking **(f)** revealed that DREADDs-expressing showed higher preference scores **(g)** (CNO day 1 vs Saline day 2: p=0.043) and reduced distance from pups **(h)** (CNO day 1 vs Saline day 2: p=0.012), after CNO injection compared to saline. Control animals expressing mCherry in CeA-LC neurons did not have significantly different pup preference **(i)** or distance to pups between saline and CNO injection **(j)** (N=6 for both groups).

We hypothesized that large pup-related responses we observed in CeA→LC neurons could be responsible for driving pup avoidance behavior in naïve virgin females. To test this, we chemogenetically suppressed CeA→LC neurons in naïve virgin female mice and measured their pup-approach behaviors. We expressed inhibitory DREADD receptor in CeA→LC neurons by injecting retrograde Cre into LC, and either cre-dependent hM4D(Gi) (for experimental animals) or cre-dependent mCherry virus (for control animals) into CeA (Fig. 3a,b). We then injected clozapine-N-oxide (CNO) before the first exposure to pups and found that naïve-virgin females expressing inhibitory DREADDs had higher pup preference score than mCherry controls (Fig. 3c,d; mCherry-naïve: 0.19±0.13 preference score, N=10, Gi-naïve: 0.58±0.37, N=13, p=0.016). Pup preference scores of DREADDs-expressing naïve virgins were not different than those of experienced females tested similarly (Fig. 3d; mCherry-experienced: 0.60±0.11, Gi-naïve: 0.51±0.22, N=4 both groups, p>0.05). To compare the behavior of the same animals over time, we repeated these experiments in a separate cohort, testing preference for pups over 4 consecutive days while alternating injecting either saline or CNO (Fig. 3e). Animals expressing inhibitory DREADDs in CeA→LC neurons had higher pup preference scores on days when CNO was injected (Fig. 3g; Saline day 1: 0.09±0.20, CNO day 1: 0.33±0.19, Saline day 2: −0.08±0.23, CNO day 2: 0.48±0.21, *N*=6, CNO day 1 vs Saline day 2: p=0.043) and spent more time interacting with pups saline (Fig. 3h; Saline day 1: 14.68±3.71% of total test time, CNO day 1: 28.71±5.71%, Saline day 2: 12.06±4.80%, CNO day 2: 45.26±10.95%, N=6, CNO day 1 vs Saline day 2: p=0.031). We did not observe similar differences between saline and CNO trials in mCherry controls (Fig. 3i,j; N=6, p>0.05). These data indicate that pup avoidance by naïve virgin females is due in part to activity of CeA→LC projections, such that activity of this projection is aversive.

### CeA oxytocin receptors are required for reducing pup aversion

In several brain regions, such as auditory cortex and MPOA, oxytocin receptors regulate sensory and social information processing and synaptic plasticity^27–29,36^. CeA oxytocin signaling has been shown to reduce freezing responses after fear conditioning. Given the high level of oxytocin receptor expression in CeA^25,26^, we hypothesized that CeA oxytocin signaling would mediate the plasticity of pup-approach behavior we observed in virgin female mice following cohousing.

**Figure 4.**
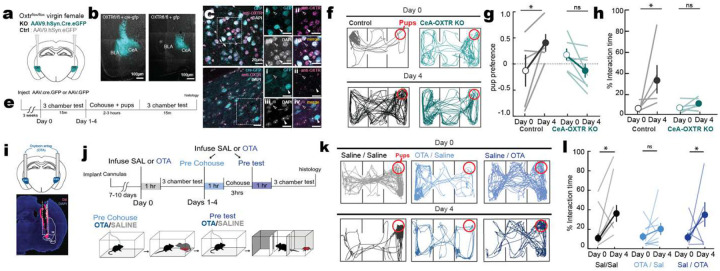
Oxytocin receptors in CeA are required for switch to pup approach **a-g,** Removal of oxytocin receptors in CeA prevented switch from pup avoidance to approach in virgin females. Schematic (**a**) and histology (**b**) of viral strategy for conditional knockdown of CeA oxytocin receptors by injecting Cre-GFP or GFP into OXTR^fl/fl^ females. Scale, 100 μm. **c,d,** Representative histology of immunohistochemical stain using anti-OXTR antibody of gfp control (**c**) and cre injected (**d**) to confirm effectiveness of knockout. Scale, 20 μm. **e,** schematic of experimental protocol including daily cohousing. **f-h,** KO of OXTR in CeA prevents increase in pup approach after cohousing. **f,** Video tracking of example control (left) and OXTR-KO (right) animals. **g,** Control mice show characteristic increase in pup preference after cohousing, but conditional knockouts did not increase preference for pups (day 0 versus 4; control: p=0.012, KO mice: p = 0.071, two-way ANOVA). Lines, individual animals; circles, mean ± s.e.m. **h**, Virgins with OXTR-KO spent more time interacting with pups after cohousing (day 0 versus 4; control: p=0.030, KO: p = 0.706, N=5 control mice, N=7 conditional OXTR-KO mice). **i-l,** Oxytocin during cohousing, but not preference test, is required for switch to pup approach. **i,** Schematic of cannula surgery (top) and histology of cannula track with DiI infusion (bottom). **j,** Illustration of infusion protocol in which OTA was delivered bilaterally into CeA either before cohousing but not preference test (OTA/Saline; N=9), before preference test but not cohousing (Saline/OTA; N=6), or neither (Saline/Saline; N=7). Only OTA/Saline animals failed to increase their approach of pups evidenced by video tracking **(k)** and had no significant increase in proportion of time spent interacting with pups, unlike Saline/Saline and Saline/OTA animals (**l**) (Sal/Sal, p = 0.011, OTA/Sal, p = 0.359, Sal/OTA, p = 0.029).

To test this, we generated a regionally-specific oxytocin receptor knockout by injecting CeA of pup-naïve virgin female OXTR^*flox/flox*^ mice with either a Cre-GFP virus or a GFP virus as a control (Fig. 4a,b). Using a custom validated antibody to the mouse oxytocin receptor^25^, we verified that oxytocin receptors were selectively deleted in the CeA of OXTR^*flox/flox*^ mice injected with the Cre virus (Fig. 4c,d). We then tested these animals on the 3-chamber pup preference assay daily for four days, cohousing for 2–3 hours per day following the initial tests on Day 0 (Fig. 4e). Animals lacking oxytocin receptors in CeA remained pup-averse throughout the four days of testing and did not show increased pup preference scores (Fig. 4g, control animals, black, day 0 mean: −0.17±0.30, day 4 mean: 0.39±0.16, N=5, p=0.02; OXTR CeA KO, blue, day 0 mean: 0.20±0.11, day 4 mean: −0.11±0.13, N=7, p=0.07) or interaction time (Fig. 4h, control animals, black, day 0 mean: 5.35±2.10 % of total test time, day 4 mean: 30.45±14.02, N=5, p=0.029; OXTR CeA KO, blue, day 0 mean: 6.47±1.68, day 4 mean: 9.70±1.98, N=7, p=0.706).

These experiments show that CeA oxytocin receptors help gate the behavioral switch from pup avoidance following cohousing, but do not indicate when oxytocin signaling is required during this process. Oxytocin could be released during cohousing to enable long-term plasticity of pup responses, which might cause an enduring reduction of avoidance in the test phase. Alternatively, oxytocin could be required during the 3-chamber preference assay to actively modulate pup-related activity during social interactions. To distinguish between these possibilities, we chronically implanted cannulae to infuse CeA bilaterally with either the specific oxytocin receptor antagonist OTA (0.5 mg/ml) or saline as a control, before cohousing or before the preference test (Fig. 4h–j). If oxytocin signaling is actively required for improved pup-approach behavior following cohousing, infusing OTA before the 3-chamber pup preference test should abolish pup preference.

However, if oxytocin is primarily required during cohousing, OTA infusion before cohousing but not preference testing should have a stronger effect. We found that increased interactions with pups were prevented by OTA infusion before cohousing, but not before preference testing (Fig. 4k,l; Saline infusion before cohousing and test (Sal/Sal), black: day 0 = 8.43±2.62% total time interacting, day 4 = 34.52±8.60% total time interacting, N=7 mice, p=0.011, OTA infusion before cohousing (OTA/Sal), light blue: day 0 = 11.18±5.06%, day 4 = 18.90±6.11%, N=9, p = 0.36, OTA infusion before 3-chamber test (Sal/OTA), dark blue, day 0 = 9.32±2.38%, day 4 = 32.89±12.73%, N=6, p=0.029). We conclude that oxytocin is released during cohousing, likely by interactions with the cohoused dam encouraging pup contact^27^, and orchestrates long term changes to pup-related activity that persistently increase pup preference.

### Oxytocin-mediated plasticity of synaptic transmission in CeA

We next sought to determine how oxytocin modulates synaptic transmission and plasticity of CeA→LC projections. Work from other labs previously established that oxytocin receptors are primarily concentrated in the lateral subdivision of the CeA and not generally found on long-range CeA projectors of the medial subdivision^23,26,31,32,37^. We therefore hypothesized that rather than expressing oxytocin receptors themselves, oxytocin may modulate activity of CeA→LC neurons through local circuit mechanisms.

**Extended Data Figure 3.**
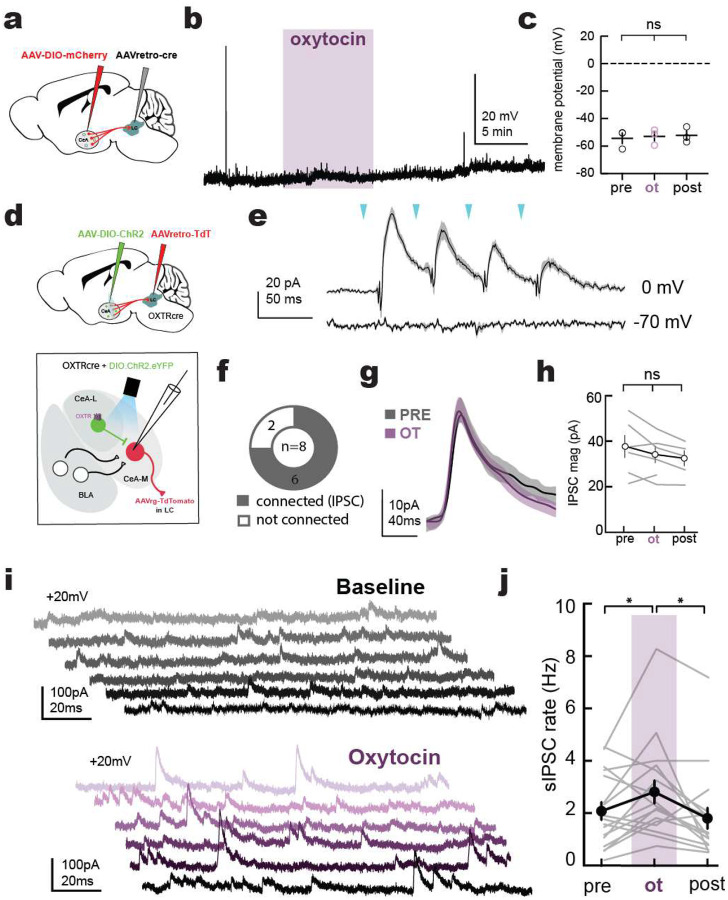
CeA oxytocin receptors modulate presynaptic inhibitory inputs to CeA→LC neurons **a**, Whole-cell recording of CeA→LC neurons expressing mCherry. Example current-clamp recording (**b**) and mean membrane potential before ‘pre’, during ‘ot’ and after ‘post’ oxytocin wash on (**c**) show that oxytocin does not depolarize CeA→LC neurons (pre vs ot: p=0.363, n=3 cells, Fisher’s test). **d-h,** Oxytocin does not modulate OXTR+ IPSCs onto CeA→LC neurons. **d**, Whole-cell recordings in CeA→LC neurons with optogenetic stimulation of OXTR+ neurons in CeA. **e**, Optogenetic stimulation of CeA-OXTR+ neurons elicited IPSCs in CeA→LC neurons. **f**, 6/8 recorded neurons received inhibitory input from CeA-OXTR+ cells. **g**, average IPSCs from example recording during the 5 minute baseline period (‘pre’, grey) and 10–15 minutes after oxytocin wash (‘ot’, purple) with shaded ± s.e.m. **h**, Average magnitude of OXTR+ IPSCs did not change with oxytocin washin (pre vs ot, post: p=0.33, n=6 cells, one-way ANOVA with Tukey’s test). **i,j**, Oxytocin increased spontaneous IPSCs onto CeA-LC neurons. **i**, Example traces of the same neuron at baseline (grey) or after oxytocin washin (purple). **j**, Average number of spontaneous inhibitory events was greater during oxytocin washin (pre vs. ot: p=0.017, ot vs. post: p=0.028, Fisher’s test).

We made whole-cell recordings from CeA neurons in brain slices, asking if CeA→LC cells responded to oxytocin. Current-clamp recordings revealed that oxytocin washin (2 μM) did not depolarize CeA→LC neurons (Extended Data Fig. 3a–c; pre: −54.29±3.93 mV, ot: −52.94±3.31 mV, post: −52.19±3.27 mV, *n*=3 cells, p=0.36), showing that they are not directly modulated by oxytocin^38,39^. Next, to determine whether CeA-LC neurons instead receive local inputs from oxytocin receptor-expressing neurons, we injected OXTR-Cre mice with a Cre-dependent channelrhodopsin 2 virus in CeA and a retrograde tdTomato virus in LC (Extended Data Fig. 3d). We stimulated OXTR+ CeA neurons with blue light while recording in voltage-clamp from CeA→LC neurons and found that CeA-OXTR stimulation resulted in large inhibitory postsynaptic currents (IPSCs) but no excitatory postsynaptic currents (EPSCs) in the majority of CeA→LC cells (Extended Data Fig. 3e,f). The magnitude of optically evoked synaptic inputs was unaffected by oxytocin (Extended Data Fig. 3g,h; **‘**pre’, baseline: 38.88±4.73pA, ‘ot’, 15–20 minutes after oxytocin: 35.53±3.54 pA, ‘post’, 25–30 minutes after oxytocin: 33.93±3.24 pA, n=6 cells, p=0.52 pre vs ot).

Despite synaptic inhibition from OXTR+ inhibitory inputs being unchanged, we found that oxytocin washin did increase the rate of spontaneous presynaptic inhibitory inputs onto CeA→LC neurons (Extended Data Fig. 3i,j; pre: 4.03±1.12 IPSCs sec^−1^, ot: 5.94±1.45 IPSCs sec^−1^, post: 4.18±1.47 IPSCs sec^−1^, n=18 cells, p = 0.018 pre vs ot, p = 0.028 ot vs post). These findings are consistent with previous work conducted in rats and male mice which has found that though CeA expresses oxytocin receptors, they are primarily concentrated on subpopulations of that provide local inhibition to long range projection neurons^26,31,32^.

**Figure 5.**
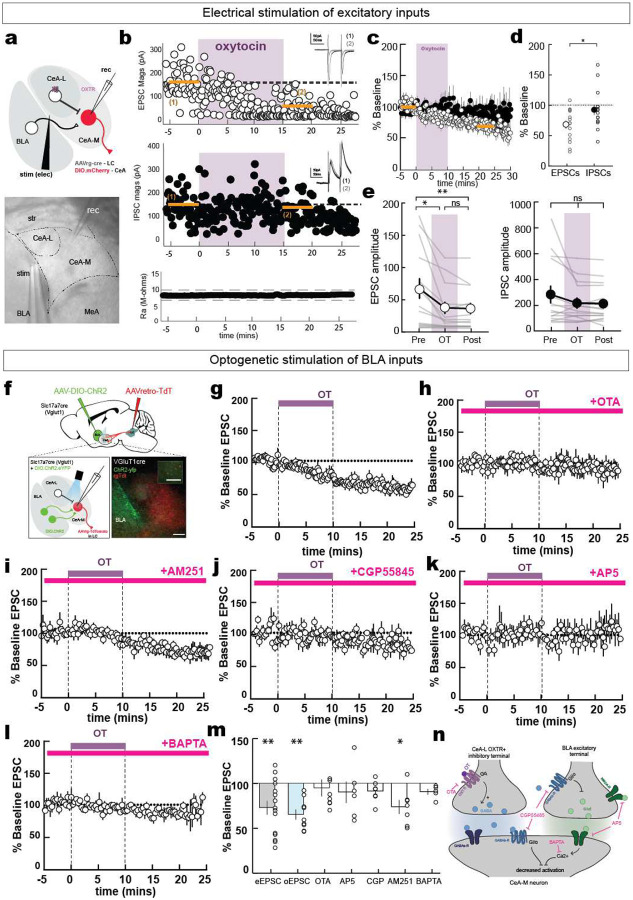
Oxytocin enables long-term excitatory depression via postsynaptic NMDA receptors **a**, Schematic (top) and DIC image of whole-cell recordings from TdTomato^+^ LC-projecting neurons in CeA with local electrical stimulation. **b-e**, Long-term depression of EPSC magnitude with oxytocin. **b**, Example cell EPSCs (top) significantly reduced after oxytocin wash with no change to IPSCs (middle) or Ra, access resistance (bottom). Insets are average currents from times marked by orange lines. **c**, Average instantaneous currents normalized to 5-minute period before oxytocin washin. **d**, Baseline-normalized postsynaptic current magnitude 15 to 20 minutes after oxytocin wash (n=15 neurons, Student’s two-tailed paired t-test). Small circles, individual cells; large circles, mean ± s.e.m. **e**, Summary of EPSC (left) and IPSC (right) magnitude changes from −5 to 0 minutes before (‘pre’), 15 to 20 minutes after (‘OT’), and 30–35 minutes after oxytocin (‘post’). EPSCs, pre versus OT p=0.0097, pre versus post p=0.0026, n=15 neurons, one-way ANOVA with Tukey’s post hoc test). **f,** Schematic for whole-cell recording of CeA→LC neurons with optogenetic stimulation of BLA inputs. Scale, 200 μm. **g-m,** Oxytocin washin during optogenetic stimulation of BLA inputs in the presence of control ACSF (**g**) (n=9) or with pre-incubation of the slice with oxytocin receptor antagonist OTA (**h**) (n=9), cannabinoid receptor antagonist AM251 (**i**) (n=7), GABAb receptor antagonist CGP55845 (**j**) (n=7), NMDA receptor antagonist AP5 (**k**) (n=6) and by addition of BAPTA to the recording pipette (**l**) (n=5). **m,** Oxytocin elicited significant LTD during simple electrical stimulation (eEPSC, n=9, p=0.019, Wilcoxon test), with optogenetic stimulation (oEPSCs, n=9, p=0.004, and with addition of AM251 (n=7, p=0.031), but not in the presence of OTA (n=8, p=0.38), AP5 (n=6, p=0.44), CGP55848 (n=7, p=0.109), or intracellular BAPTA (n=5, p=0.063). **n,** Mechanisms for NMDA and GABAb receptor-dependent LTD induced by oxytocin in CeA.

Having established that CeA-LC neurons were not directly modulated by oxytocin receptors, we asked how oxytocin affected synaptic transmission in CeA. To do this, we first used electrical stimulation of local excitatory and inhibitory inputs CeA-LC neurons (Fig. 5a).

Although oxytocin caused a significant increase in spontaneous inhibitory synaptic currents in CeA-LC neurons (Extended Data Fig. 3e,j), inhibitory synaptic input magnitude was not affected by oxytocin (Fig. 5b,c). Instead, we observed a significant long-term depression (LTD) of the magnitude of EPSCs, which persisted for up to 30 minutes after oxytocin washin (Fig. 5d–f; ‘Pre’, EPSC magnitude baseline: 68.0±15.8 pA, ‘OT’, 15 minutes after addition of oxytocin: 40.97±10.20 pA, p =0.0097; ‘Post’, 25–30 minutes after oxytocin: 36.51±8.78 pA, p=0.0026, n=15 cells, Fig. 5e).

How can oxytocin receptors, primarily located on local presynaptic inputs to CeA→LC neurons, help induce LTD of excitatory transmission? To determine the mechanisms of this form of LTD, , we examined excitatory inputs from the basolateral amygdala (BLA) which provides dense excitatory input to the CeA preferentially excited by aversive stimuli^40–43^. We used virgin female Slc17a7(VGlut1)-Cre mice injected with Cre-dependent channelrhodopsin-2 to obtain specific optical access to BLA principal neurons (Fig. 5g). We also injected these mice with a retrograde tdTomato virus in the LC to identify CeA→LC neurons in slices. We recorded optically-evoked excitatory postsynaptic currents (oEPSCs) in CeA→LC neurons and found that oxytocin washin caused a significant decrease in the magnitude of BLA-oEPSCs in CeA-LC neurons (OT-LTD) (Fig. 5g; p=0.004). This LTD was prevented by pre-incubating slices with OTA (2 μM), confirming that oxytocin receptors are required for this effect (Fig. 5h; p=0.38).

Excitatory LTD could be induced by several factors. We first asked whether endocannabinoid signaling contributed to OT-LTD as type 1 cannabinoid receptors (CB1Rs) are found on BLA terminals in CeA where they have been shown to mediate both excitatory and inhibitory plasticity^44^. However, CB1 blockade with AM251 (2 μM) did not prevent a significant reduction in oEPSC magnitude (Fig 5i; p=0.031). Given our findings that oxytocin causes increased spontaneous inhibition onto CeA-LC neurons, we next wondered whether this increased GABA release could play a part in OT-LTD at BLA-CeA synapses. Specifically, we examined the involvement of GABA-B receptors (GABAbR) as previous studies have shown that GABAbR activation in CeA is sufficient to induce presynaptic LTD and impair fear conditioning^45,46^. We incubated slices with the specific GABAbR blocker CGP-55845 (5μM) before oxytocin and found that this did prevent oxytocin-dependent LTD (Fig. 5j; p=0.11, Wilcoxon test).

We then asked whether N-methyl-D-aspartate receptors (NMDARs) were required for OT-LTD^47,48^. We blocked NMDARs using the antagonist AP5 (50 μM) and found that this prevented OT-LTD (Fig. 5k; p=0.43). Furthermore, adding the calcium chelator BAPTA (10 mM) to the patch pipette to prevent the Ca^2+^-based signal transduction cascades blocked OT-LTD, suggesting a postsynaptic mechanism (Fig. 5l).

We therefore conclude that oxytocin elicits LTD of excitatory BLA inputs onto CeA-LC neurons through a postsynaptic mechanism that depends both on GABAb and NMDA receptors (Fig. 5m). While it is unclear whether these two receptor types are exerting their effects in parallel or in concert, we hypothesize that LTD at BLA-CeA-LC synapses could function in a manner similar to what has been reported in the neocortex^49^, where coincident activation of both NMDA and another type of Gi/o coupled receptor is required for excitatory LTD (Fig. 5n).

### Modulation of LC firing by CeA inputs

**Figure 6.**
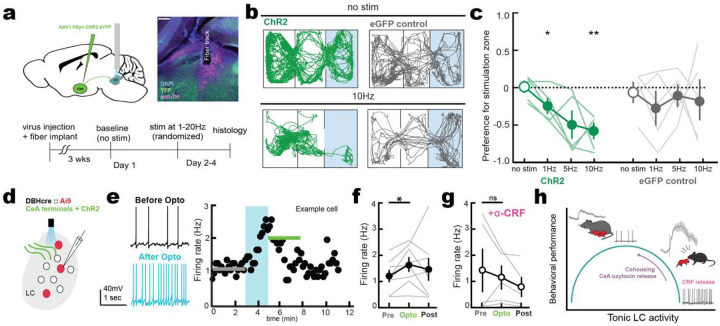
CeA→LC projections release CRF to increase LC firing and induce aversion **a-c**, Optogenetic stimulation of CeA terminals in LC. a, schematic (top) and timeline (bottom) of optogenetic stimulation experiments of CeA terminals with fibers implanted over bilateral LC. **b**, representative tracking for animals expressing channelrhodopsin-2 (green) or eGFP control (grey) with no stimulation (left) or receiving 10Hz stimulation (right) over bilateral LC when in right section. **c**, stimulation of CeA inputs in LC drives real-time place aversion (*N* = 6; no stim versus 1 Hz ChR2, p=0.020; no stim versus 10 Hz, p=0.002, one-way ANOVA with Dunnett’s correction for multiple comparisons). **d-g**, In vitro recordings of LC neurons with CeA input stimulation. d, schematic of surgery and whole cell slice recording of identified noradrenaline neurons in slices. **e**, example neurons showing firing of LC neurons before (black) and after (blue) optogenetic stimulation of CeA inputs. Firing rate increase in LC neurons during optogenetic stimulation (**f**) is blocked by addition of CRF-receptor antagonist ∝-helical CRF (**g**) (n=8 cells, p=0.013, Student’s paired two-tailed t test). **h,** Model of CeA activation of LC for maternal behavior. Pup interactions trigger large responses in CeA-LC projections of pup-naïve virgins, resulting in CRF release and high firing rates in LC neurons. During cohousing when oxytocin is released, these responses are diminished such that LC neurons can fire in the medium tonic range and animals can be attentive and task engaged during caregiving.

If pup-related activity in CeA-LC projections is indeed driving pup avoidance in naïve virgin female mice, stimulating this input should result in aversion or avoidance. To test this, we injected a virus expressing either channelrhodopsin-2 or eGFP bilaterally into CeA and implanted optical fibers over LC (Fig. 6a). We found blue light stimulation of CeA-LC terminals expressing channelrhodopsin-2 but not eGFP controls induced real-time place aversion of the stimulated section of the chamber (Fig. 6b,c; preference score for stimulated chamber for ChR2-expressing mice, no stim: 0.013±0.062; 1 Hz: −0.24±0.15, p=0.020; 5 Hz: −0.50±0.15, p=0.077; 10 Hz: −0.55±0.083, p=0.0018; eGFP-expressing, no stim: −0.061±0.13; 1 Hz: −0.27±0.25, p=0.51; 5 Hz: −0.11±0.14, p=0.71; 10 Hz: −0.11±0.25, p>0.9; ChR2, N=6 mice, eGFP, N=5 mice).

To determine how CeA inputs modulate LC firing, we performed whole-cell current-clamp recordings from LC noradrenaline cells (Fig. 6d). We found that stimulating CeA afferents augmented spontaneous firing (Fig. 6e,f; n=6 cells, p=0.0127). Consistent with previous studies conducted in male mice^50^, this effect was blocked by the addition of the corticotropin releasing factor (CRF) receptor antagonist, alpha-helical CRF (Fig. 6g; n=5 cells, p=0.55, Fischer’s test). Taken together, these results suggest that contact with pups in inexperienced pup-naïve virgin female mice causes a CRF-dependent increase in LC noradrenaline neuron firing which is aversive and leads avoidance of pups.

## Discussion

We identified here a neural circuit mechanism that transforms pup aversion into attention. We believe that the dynamics of the CeA→LC projections might have important consequences for social interactions, in some cases ethological and perhaps in other cases pathological. Some strains or species of mice live in large colonies often with siblings and are exposed to pups and effective parental behaviors from an early age. This would likely provide opportunities for the mechanisms of plasticity described here to reduce LC activation in the presence of related or unrelated infants. In other cases, however, it might be more evolutionarily advantageous to ignore or avoid pups as a non-parental adult, for example to avoid investing resources in unrelated neonates or to escape from the territory of other potentially aggressive adults. Once an animal becomes a parent, failure to reduce LC activation might have disastrous consequences for infants and family members, leading to infant neglect and postpartum conditions impacting parental care such as anxiety or depression^1,27–30^. This could happen if oxytocin levels are not sufficient to downregulate CeA excitatory inputs, or if CeA or LC activity remains high due to other stress pathways or inputs engaging these brain areas.

CeA is a complex hub that seems to route sensory information to different behavioral state modulatory areas depending on the computation of valence. For this reason, we used a projection-specific monitoring approach, rather than labeling specific cells identified by molecular markers as most CeA cell types seem to have multiple projection targets^30^. The local circuitry of CeA consists largely of mutually inhibitory GABAergic cells, and we hypothesize that the reduction in output to LC might also have consequences for enabling increased activity in other subnetworks or projections, e.g., to MPOA or BNST, although likely in a subtle manner beyond the detection limit of fiber photometry used here. It is generally believed that performance on many tasks follows an inverted U-shaped curve of LC activity, such that if LC activation is too low or too high, behavioral performance is suboptimal. We hypothesize that reducing CeA-LC input brings arousal to a more optimal level for attending to infants, reducing stress and avoidance and then permitting maternal circuits such as NAc or MPOA to lead to appropriate childcare.

## Methods

### Animals

All procedures were approved under NYU School of Medicine Institutional Animal Care and Use Committee (IACUC) protocols, in accordance with National Institutes of Health (NIH) guidelines. Mice were housed in fully equipped facilities in the NYU School of Medicine Science Building. The facilities were operated by the NYU Division of Comparative Medicine. All mice were bred and housed in our facility kept at ~22 °C, with a relative humidity of ~45%. The light cycle was 12 h on–12 h off (06:30–18:30 light). Wild-type C57BL/6N (Taconic, B6-F) mice were used for projection-specific fiber photometry, chemogenetic inhibition, and cannula infusion experiments. Oxtr^*flox*^ (strain 008471), Oxtr^*cre*^ (strain 031303), DBH^cre^ (strain 0.33951), Ai9 (strain 007909), and Vglut1^cre^ (strain 023527) mice were purchased from the Jackson Laboratories. All test mice were between 8–15 weeks at the time of behavioral recording and over 8 weeks at the time of surgery. All pups used for testing were under P10 and bred in-house from wild-type breeders.

### 3-chamber social approach assay

3-chamber social approach assays were designed following standardized protocols^1–3^. Custom behavior boxes (30 cm width × 30 cm height × 60 cm length) were designed and assembled with 1/4” acrylic. Animals were given one 15-minute habituation session for the arena on the day prior to recording. On experimental days, animals followed a standardized paradigm consisting of a 10-minute habituation to the center chamber followed by a sociability test where animals were allowed to explore the full arena and interact with social and non-social targets. Videos were recorded using e3Vision cameras (White Matter) and processed using the open-source video analysis pipeline ezTrack^4^ and analyzed with custom MATLAB code. Pup preference scores were tabulated by calculating (time in pup chamber – time in object chamber)/(time in pup chamber + time in object chamber) resulting in a score of −1.0 (denoting total pup avoidance) to 1.0 (denoting complete preference for pups). Interaction times were calculated by measuring the number of total video frames during which the test animal was positioned directly above the pups, divided by the total number of frames analyzed to determine relative fraction of total time that each animal interacted with pups. Distance from pups was measured by summing the instantaneous distance between the center of mass of the test animal and the center of the cluster of pups, then dividing this by the total number of frames to achieve an average distance from the pups over the course of the entire 15 minute test period. Experimenters were blinded to subject group during analysis.

### Cohousing

For testing the effects of cohousing experience on pup-directed sociability, animals were divided into cohoused or non-cohoused groups. Cohoused animals were placed into a cage with a nursing dam and litter of pups <P10 for 2–3 hours daily following the baseline assessment tests on day 0. Animals in the non-cohoused group were placed in a cage with an age matched unfamiliar female mouse for the same period. Animals were then placed back into their home cage for 1-hour preceding behavioural assays.

### Retrieval

Retrieval ability was measured after sociability testing in the home cage. Animals were given 2-minute blocks to collect scattered pups back to the nest before time out between blocks. 5 pups were scattered during each block and the total number of pups collected over both blocks was used as the retrieval score out of a possible 10 total pups. Retrieval sessions were filmed, allowing for measurements of retrieval latency and duration post-hoc.

### Stereotaxic surgeries

Mice were anesthetized with 1–1.5% isoflurane and placed in a stereotaxic apparatus. Viruses were delivered to brain regions through glass pipettes using a Nanoject III (Drummond Scientific, 3-000-207). Viruses were delivered at a speed of 5nl sec^–1^ and between 50 and 250nl of virus was delivered during each injection. We used the following stereotaxic coordinates (in mm: AP, anteroposterior; ML, mediolateral; DV, dorsoventral): CeA (−0.975 AP, +/−2.65 ML, −4.2 DV), LC (−5.3 AP, +/− 0.85 ML, −3.4 DV), LH (−1.1 AP, +/−1.1 ML, −4.7 DV), BNST (+0.3 AP, +/−0.9 ML, −4.0 DV), BLA (−1.5 AP, +/−3.0 ML, −4.4 DV).

### Fiber photometry implantations

For projection specific targeting of CeA populations, wild-type mice were injected with AAVrg-hSyn-Cre-WPRE-hGh (Addgene, 105553-AAVrg, 7×10^12^ vg/mL) in in LC, LH, and BNST and with AAV9-syn-FLEX-jGCaMP7s-WPRE (Addgene, 104491-AAVrg, 2.5×10^12^ vg/mL) in CeA ipsilateral to cre injection. 400-μm optical fibre (Thorlabs, CFMC54L05) were then implanted 100 μm above the injection site. Fibers were secured to the skull using C&B Metabond dental cement (Parkell). Experiments were performed unilaterally on both left and right sides with cre and GCaMP viruses injected on ipsilateral hemispheres in each experiment to minimize off-target labeling. Experiments were performed 3–4 weeks after surgery.

### Photometry

Mice were tethered to an optical fiber (Thorlabs) which was used to deliver a 400 Hz blue light (40–45 μW) generated by a 470 nm LED (Thorlabs, M470F1) connected to a LED driver (Thorlabs, LEDD1B) to excite GCaMP7s. A 405 nm LED (Thorlabs, M405FP1) connected to another LED driver was used to deliver the isosbestic control light at 330 Hz (10–15 μW) through the same fiber. Light passed through 405 nm and 469 nm excitation filters and dichroic mirrors then to the brain. Emitted light passed back through the same fibre via a dichroic mirror, 525 nm emission filter, and adjustable zooming lens (Thorlabs, SM1NR05) before being detected by a femtowatt silicon photoreceiver (Newport, 2151). Digital signals were then collected using an RX8 Multi-I/O Processor and Active X Software (Tucker-Davis Technologies). The analog readout was low-pass filtered at 10 Hz.

### Behavioural testing during photometry

Animals were placed in a clean 40 cm × 40 cm behavioral arena and allowed to habituate for 30 minutes prior to all recordings. Animals were then tethered to the fiber optic patch cable and recorded for a baseline period of 5–10 minutes in the dark which was subsequently used to normalize the fluorescence signal. Photometry sessions were video recorded at 30 fps using e3Vision cameras (White Matter) and behavioral epochs were hand scored using Datavyu software. Animals were presented with various social and non-social stimuli during the same recording sessions. After 1–2 baseline recordings some animals were then cohoused with dams nursing litters <P10 for 4–7 days and then retested.

### Analysis of photometry signals

The 470 nm GCaMP Ca2+ signal was normalized to the 405 nm isosbestic signal using a linear regression model to generate *F*_0_, the normalized fluorescence predicted from a first-order polynomial fit during the baseline period. The fluorescence signal was then converted into Z-score for analysis as *z* = *(F*_0_ − *μ)/σ* where *μ* and *σ* represent the mean and standard deviation of *F*_0_ during the baseline period respectively. The baseline period refers to the 5 minute period preceding the first behavioral test. Z-score analysis was also performed using the entire length of the recording trace and resulting values and statistical tests were unchanged. Behavioral epochs were manually scored using Datavyu software and custom MATLAB code was used to align epochs to fluorescence signals. To calculate the magnitude of fluorescence responses to individual behaviors, onsets were set as time 0 and the mean fluorescence change from −5 s and 0 was subtracted from to the mean signal from 0–5 s. Onsets of adult and pup interactions were determined as the video frame during which the edge of the test animal’s nose contacts the edge of the outline of the investigation target as viewed from above.

### Chemogenetics

Virgin female mice were injected bilaterally LC with retrograde Cre (AAVrg-hSyn-Cre-WPRE-hGh, Addgene, 105553-AAVrg, 7×10^12^ vg/mL) and in CeA with Cre-dependent mCherry (AAV8-hSyn-DIO-mCherry, Addgene, 50459-AAV8, 2.5×10^12^ vg/mL) for controls or Cre-dependent hM4Di (AAV8-hSyn-DIO-hM4D(Gi)-mCherry Addgene, 44362-AAV8, 2.5×10^12^ vg/mL) for chemogenetic inhibition. For initial preference testing, experiments were conducted similar to previous lesion studies^5^ by first injecting virgins with saline and determining their preferred section of the 3-chamber arena. On the next day, virgins were injected with 1 mg kg−1 CNO 40–45 minutes before being placed in the center of the arena for a 10 minute habituation period and 15 minute preference test. The following day, pups were added to the subject animal’s previously preferred section and the test was repeated. Both pup-naïve virgins and animals that had been cohoused for over 1 week (experienced virgins) were used for initial preference test experiments. For 4-day chemogenetic experiments, only pup-naïve virgin females were used. Virgins expressing hM4D(Gi) or mCherry in CeA-LC neurons (as described above) were injected with either 1 mg kg−1 CNO or the equivalent volume of saline each day for 4 days 40–45 minutes before preference testing. Preference testing was conducted as described above.

### Cannula implantation and infusion

Mice were implanted with infusion cannula (26 gauge, 5 mm, Plastics One, C315GAS-5/SPC) bilaterally over the CeA and outfitted with custom dummy cannulas and dust caps (Plastics One, C315DCS-5/SPC) to prevent cannula obstruction. Experiments were performed 7–10 days after surgery. For intracerebral infusion experiments animals were given 1 μl of the OXTR antagonist OTA ((d(CH2)51,Tyr(Me)2,Thr4,Orn8,des-Gly-NH29)-vasotocin trifluoroacetate salt, Bachem, 4031339) at 0.5 mg ml−1 (0.5 mg total per hemisphere) or an equal volume of sterile saline over CeA. Liquid was infused over 2 min with a Pump 11 Elite Syringe Pump (Harvard Apparatus, HA1100). Animals were placed into three groups: Saline/Saline, OTA/Saline, or Saline/OTA referring to the infusion they received prior to cohousing and prior to preference testing, respectively. Animals were tested 1 hour after infusion to reach maximum oxytocin receptor antagonism^6^.

### Oxytocin receptor conditional knockout

To knockout OXTRs in CeA, we bilaterally injected CeA of Oxtr^*flox*^ virgin female mice with virus expressing cre tagged with GFP (AAV9-hSyn-HI-eGFP-cre-WPRE, Addgene, 105540, 1×10^12^ vg/mL), or GFP alone (AAV9-hSyn-eGFP, Addgene, 50465, 1×10^12^ vg/mL) for controls. Virus was allowed to express for 3 weeks before animals went through daily cohousing and preference testing as previously described. Videos were recorded using e3Vision cameras (White Matter) and processed using the open-source video analysis pipeline ezTrack^4^ and analyzed with custom MATLAB code. Experimenters were blinded to subject group during analysis.

### Whole-cell recordings in brain slices

Animals were deeply anesthetized by isofluorane inhalation and perfused with ice-cold sucrose cutting solution containing 87 mM NaCl, 75 mM sucrose, 2.5 mM KCl, 1.25 mM NaH_2_PO_4_, 0.5 mM CaCl_2_, 7 mM MgCl2, 25 mM NaHCO_3_, 1.3 mM ascorbic acid and 10 mM D-glucose, bubbled with 95%/5% O_2_/CO_2_ (pH 7.4). The brain was extracted and 250 μm slices were prepared with a vibratome (Leica P-1000) while being submerged in the same ice-cold cutting solution. Slices were rapidly placed in warm artificial cerebrospinal fluid containing 124 mM NaCl, 2.5 mM KCl, 1.5 mM MgSO_4_, 1.25 mM NaH_2_PO_4_, 2.5 mM CaCl_2_, 26 mM NaHCO_3_ and 10 mM d-glucose bubbled with 95%/5% O_2_/CO_2_ (pH 7.4) and maintained at 33–35 °C for 30 min. Slices in ACSF were then removed from head and maintained at room temperature for at least 1 hour before use.

Slices were transferred to the recording chamber and superfused with ACSF (2.5–3 ml min^−1^) at 33 °C. Slices were visualized using an Olympus 40× water-immersion objective. Somatic whole-cell voltage-clamp or current-clamp recordings were made using Multiclamp 200B or 700B amplifier (Molecular Devices) using Pipettes with resistances between 3–7 MΩ made of borosilicate glass capillaries with an outer diameter of 1.5 mm and an inner diameter of 0.86 mm (Sutter, BF-150-86-10). Data were filtered at 2 kHz, digitized at 10 kHz, and acquired with Clampex 10.7 (Molecular Devices). Data were analyzed using custom-written Matlab code (MathWorks) and Clampfit 10.7 (Molecular Devices). For voltage clamp experiments, access resistance was calculated with brief current steps. For current clamp recordings, access resistance was measured with a membrane test periodically during recording. Recordings were excluded from analysis if the access resistance (Ra) changed by more than 30% compared to baseline.

For voltage clamp recordings, electrodes were filled with intracellular solution containing 130 Cs-methanesulfonate, 1 QX-314, 4 TEA-Cl, 0.5 BAPTA, 4 MgATP, 0.3 Na-GTP, 10 phosphocreatine, 10 HEPES (osmolality, 280 mOsm; pH 7.4 adjusted with KOH). For current clamp recordings, pipettes were filled with solution containing 127 mM potassium gluconate, 8 mM KCl, 10 mM phosphocreatine, 10 mM HEPES, 4 mM magnesium ATP and 0.3 mM sodium GTP (osmolality, 285 mOsm; pH 7.2 adjusted with KOH).

For experiments with electrical stimulation to assess the effects of oxytocin on synaptic transmission in CeA, voltage-clamp recordings were acquired from CeA-M neurons identified by their expression of mCherry in animals injected with retrograde cre (AAVrg-hSyn-Cre-WPRE-hGh, Addgene, 105553-AAVrg, 7×10^12^ vg/mL) in LC and cre-dependent mCherry (AAV8-hSyn-DIO-mCherry, Addgene, 50459-AAV8, 2.5×10^12^ vg/mL) in CeA. Focal extracellular stimulation was applied with a 0.015 inch silver-chloride filaments inserted into a borosilicate theta capillary glass with an outer diameter of 1.5 mm and an inner diameter of 1.0 mm (Warner Instruments, TG150–4) filled with ACSF. Stimuli were delivered for 0.1 ms, 3–700 μA, at a rate of 2–4 stimuli every 20 seconds (with 50 ms inter-stimulus intervals). Cells were held at −70mV to assess EPSCs and then stepped to 0 mV to assess IPSCs. Bipolar glass was placed lateral to target neurons near basolateral and CeA-L nuclei to reliably evoke both excitatory and inhibitory local currents in the same neurons. Once EPSC and IPSC magnitude and access resistance was stable for 5–10 minutes, Oxytocin (1μM; Tocris 1910) in ACSF was washed on for 10–15 minutes followed by washout with control ACSF. For analysis, the baseline ‘pre’ period was designated as the 5 minutes immediately before oxytocin washin, the oxytocin ‘OT’ period as the 5-minute period 15–20 minutes after oxytocin washin began, and the ‘post’ period as the 5 minutes 25–30 minutes after oxytocin washin.

For experiments with optogenetic stimulation and measuring plasticity of BLA inputs, Vglut1^cre^ virgin females were injected with cre dependent channelrhodopsin-2 (AAV9-EF1a-fl/fl-hChR2(H134R)-EYFP-WPRE-HGHpA, Addgene, 20298-AAV9, 2.5×10^12^) in BLA and retrograde tdTomato virus (AAVrg-hSyn-mCherry, Addgene, 114472-AAVrg, 7×10^12^ vg/mL) in LC. Whole-cell voltage clamp recordings were made from CeA-LC neurons identified by their expression of tdTomato visualized with a TRITC filter. Brief blue light pulses were delivered to stimulate BLA inputs (1-ms pulses, 2 pulses per sweep, 20sec sweep, 50ms ISI). (d(CH2)51,Tyr(Me)2,Thr4,Orn8,des-Gly-NH29)-vasotocin trifluoroacetate salt (OTA; 2 μM; Bachem, 4031339), DL-2-amino-5-phosphono-pentanoic acid (AP5; 50 μM; Tocris, 0105), and Oxytocin (1μM; Tocris 1910) were dissolved directly in the extracellular solution and bath applied. Picrotoxin (50 μM; Millipore Sigma, P1675), 1-(2,4-Dichlorophenyl)-5-(4-iodophenyl)-4-methyl-N-1-piperidinyl-1H-pyrazole-3-carboxamide (AM251, 2μM, Millipore-sigma, A6226), and (2S)-3-[[(1S)-1-(3,4-Dichlorophenyl)ethyl]amino-2-hydroxypropyl] (phenylmethyl) phosphinic acid hydrochloride (CGP-55,845; 5 μM Tocris, 1248) were dissolved in DMSO and added to the extracellular solution such that the final concentration was <0.1%. 1,2-Bis(2-aminophenoxy) ethane-N,N,N′,N′-tetraacetic acid (BAPTA, 10 mM, Millipore Sigma, A1076) was first dissolved in DMSO then added to voltage-clamp intracellular solution such that the final concentration of DMSO was <0.1%. oEPSCs were all recorded in the presence of picrotoxin.

For optogenetic stimulation of CeA terminals in LC, current-clamp recordings were performed in DBH^cre^::Ai9 animals and LC noradrenergic neurons were identified by their expression of tdTomato. α-helical CRF (9–41) (5 μM Tocris, 1184) was dissolved directly into extracellular solution and bath applied. ChR2-expressing CeA terminals were stimulated with blue light pulses (1-ms pulses, 20Hz, 2 minutes). For analysis, the baseline ‘pre’ period was designated as the 3 minutes immediately before stimulation, the oxytocin ‘opto’ period as the 3-minute period immediately following stimulation, and the ‘post’ period as the 3 minutes 5–10 minutes after stimulation.

### In vivo optogenetic stimulation

For optogenetic stimulation of CeA terminals in LC in vivo, channelrhodopsin-2 (AAV9-hSyn-hChR2(H134R)-EGFP, Addgene, 26973-AAV9, 2.5×10^12^ vg/mL), or GFP (AAV9-hSyn-eGFP, Addgene, 50465, 1×10^12^ vg/mL) for controls, was injected bilaterally into CeA of wild-type virgin females. 5 mm long optic fibers (200 μm core, Thorlabs CFMLC12L05 and homemade, 0.39 NA) were then implanted bilaterally above LC at an angle of +/− 10° from center and at a depth of 0.3 mm. Fibers were secured to the skull using C&B Metabond dental cement (Parkell). After allowing 3–4 weeks for viral expression and recovery, mice were habituation to the patch cable (200 μm core, 0.39 NA, FC/PC; ThorLabs; BFYL2S01). Virgins were then tested in 3-chamber behavioral arena and blue light was delivered when virgins entered one section of the arena through the patch cable (473 nm wavelength, 1–3 mW mm^−2^) at 0,1,5, or 20 Hz, with the order of stimulation randomized. Video was collected and analyzed with the same methods as described above.

### Histology and imaging

Mice were injected (0.1 ml per 10 g) intraperitoneally with a ketamine-xylazine mixture and transcardially perfused with phosphate-buffered saline (PBS) followed by 4% paraformaldehyde (PFA) in PBS. Brains were kept in 4% PFA overnight then immersed in PBS. Brains were then sectioned at 50-μm thickness on a vibratome (Leica VT 1000-S). Sections were blocked with 3% normal donkey serum (NDS) and permeabilized with 0.1% Triton X-100 for 2 hours at room temperature on a shaker. Primary antibodies were added to 3% NDS and incubated overnight at 4°C on a shaker. Sections were then incubated with for 2–4 h at room temperature in secondary antibodies diluted at 1:500 in PBS, and finally with Hoechst 33342 (Thermo Fisher Scientific, H3570) at 1:1,000 for nuclear staining. Sections were washed with PBS (3 × 10 min at room temperature) between each incubation. Slides were mounted and coverslipped with fluoromount (Southern Biotech, 0100–01) after drying. Tissue was then examined with 10 or 20x images collected on an Olympus VS120 Automated slide scanner using OlyVIA software, and higher magnitude confocal images were acquired using a Zeiss LSM 800 with the ZEN 2.3 system. Primary antibodies used were rabbit- anti OXTR-2 (generated in-house^7^, 1:1000), chicken anti-TH (Aves Labs, 1:1000), guinea pig anti-cfos (Sysy Antibodies, 226 308, 1:4000). Secondary antibodies were donkey anti-guinea pig 647 (Jackson Immuno Research, 706-605-148, 1:500), donkey anti-rabbit 488 (Jackson Immuno Research, 115-45-003, 1:500), donkey anti-chicken 647 (Jackson Immuno Research, 703-605-155).

### Statistics

Sample sizes were not pre-determined using statistical methods but were chosen to minimize animal usage and were comparable to previous publications^8,9^. Behavioral experiments were done in one or two groups with matched numbers of control and test animals and data was combined for final analysis. Visualization of photometry and initial processing of electrophysiology data was performed using MATLAB 2024a using custom written code. All statistical tests throughout study were run using GraphPad Prism10 (GraphPad Software). All statistical analyses were two-tailed. We used 1 and 2-way ANOVAs with Dunnett’s post-hoc multiple comparisons correction in Figs. 1d,f,h,j, 6c, and Extended Data Figs. 1c,e and Tukey’s correction for Figs. 4l, 5d,e and Extended Data Figs. 3c,h. We used Fisher’s test for Figs. 1e,g,i,k, 2b,h,k,o,t, 3d,g,h,I,j, 4g,h, and Extended Data Figs. 1d,f, and 3j. We used Student’s two-tailed t-tests for Figs. 6f,g and Extended Data Fig. 2f. We used a one-sample Wilcoxon Signed Rank test for Fig. 5m. With two-way ANOVAs, post-hoc comparisons were performed depending on the recommendation in Prism. All sample sizes and definitions are presented in the figure legends. All bars throughout this study represent ±s.e.m. *, p<0.05, **, p<0.01.

## Extended Data
